# Butane-2,3-dione bis­[(4-bromo­benzyl­idene)hydrazone]

**DOI:** 10.1107/S1600536810014790

**Published:** 2010-04-28

**Authors:** Sai-Ming Yang, Yue-Tong Xu, Bang-Hua Zhang

**Affiliations:** a College of Population, Resources and Environment, Shandong Normal University, Jinan 250014, People’s Republic of China

## Abstract

The title compound, C_18_H_16_Br_2_N_4_, is a linear double Schiff base compound having two parallel 4-bromo­phenyl groups connected across a crystallographic inversion centre by flexible C—C and C=N—N=C bonds and stabilized in the solid state by weak inter­molecular Br⋯Br inter­actions [3.7992 (11) Å], generating an infinite two-dimensional network structure.

## Related literature

As a result of their geometry, including the zigzag conformation of the spacer moiety (C—C and C=N-N=C) between the two terminal groups, double Schiff base compounds have proved to be very versatile in their ability to form novel frameworks by self-assembly reactions with metal salts, see: He *et al.* (2008 ▶). For Br⋯Br inter­actions, see: Metrangolo *et al.* (2005 ▶).
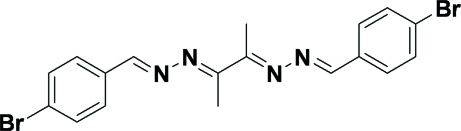


## Experimental

### 

#### Crystal data

C_18_H_16_Br_2_N_4_
*M* *_r_* = 448.17
Monoclinic, 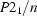

*a* = 6.9139 (16) Å
*b* = 4.0931 (10) Å
*c* = 31.480 (7) Å
β = 95.186 (3)°
*V* = 887.2 (4) Å^3^
*Z* = 2
Mo *K*α radiation
μ = 4.58 mm^−1^
*T* = 298 K
0.15 × 0.14 × 0.14 mm

#### Data collection

Bruker SMART CCD area-detector diffractometer
4238 measured reflections
1627 independent reflections
1308 reflections with *I* > 2σ(*I*)
*R*_int_ = 0.035

#### Refinement

*R*[*F* ^2^ > 2σ(*F* ^2^)] = 0.034
*wR*(*F* ^2^) = 0.081
*S* = 1.04
1627 reflections
110 parameters
H-atom parameters constrained
Δρ_max_ = 0.36 e Å^−3^
Δρ_min_ = −0.37 e Å^−3^

### 

Data collection: *SMART* (Bruker, 2000 ▶); cell refinement: *SAINT* (Bruker, 2000 ▶); data reduction: *SAINT*; program(s) used to solve structure: *SHELXS97* (Sheldrick, 2008 ▶); program(s) used to refine structure: *SHELXL97* (Sheldrick, 2008 ▶); molecular graphics: *SHELXTL* (Sheldrick, 2008 ▶); software used to prepare material for publication: *SHELXTL*.

## Supplementary Material

Crystal structure: contains datablocks I, global. DOI: 10.1107/S1600536810014790/zs2037sup1.cif

Structure factors: contains datablocks I. DOI: 10.1107/S1600536810014790/zs2037Isup2.hkl

Additional supplementary materials:  crystallographic information; 3D view; checkCIF report

## Figures and Tables

**Fig. 1.**
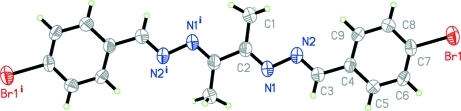
The molecular structure and atom numbering scheme for (I) with displacement ellipsoids drawn at the 50% probability level. For symmetry code (i): -x, -y+1, -z].

**Fig. 2.**
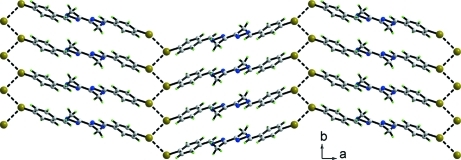
The two-dimensional network structure showing the weak Br···Br interactions as dashed lines.

## supplementary crystallographic information

### Comment

Double Schiff-base compounds, due to their specific geometry, including the zigzag conformation of the spacer moiety (C—C and C═N-N═C) between the two terminal groups, has proven to be very versatile in its ability to form novel frameworks by self-assembly reactions with metal salts (He *et al.*, 2008). In these compounds, the central C—C and N—N bridges are rotationally flexible and the significance of the relative orientations of these terminal groups in self-assembly reactions has become a matter of increasing interest in recent literature.

The structure of the title compound, C_18_H_16_Br_2_N_4_ (I) (Fig. 1) shows two parallel 4-bromophenyl groups connected by flexible C—C and C═N-N═C bonds, with the molecule having crystallographic inversion symmetry. All atoms in the molecule are coplanar resulting in a linear conformation. In the solid state, the title compound is stabilized by weak intermolecular Br···Br interactions [3.7992 (11) Å] (Metrangolo *et al.*, 2005), linking the molecules down the *b* axial direction in the cell, generating an infinite two-dimensional network structure (Fig. 2).

### Experimental

A mixture of 2,3-butanedione dihydrazone (0.57 g, 5.0 mmol) and 4-bromobenzaldehyde (1.85 g, 10.0 mmol) with 2 drops of formic acid in ethanol (60 ml) was stirred at room temperature for *ca.* 1 hour to generate the title compound as a yellow solid (2.15 g, 96% yield). Single crystals suitable for X-ray analysis were grown in dichloromethane by slow evaporation at room temperature.

### Refinement

All non-hydrogen atoms were refined with anisotropic displacement parameters. Hydrogen atoms were placed in idealized positions and treated as riding, with C—H = 0.93 Å (CH), 0.96 Å (CH_3_) and *U*_iso_(H) = 1.2 *U*_eq_(CH) and *U*_iso_(H) = 1.5 *U*_eq_(CH_3_).

### Crystal data

**Table:**

C_18_H_16_Br_2_N_4_	*F*(000) = 444
*M**_r_* = 448.17	*D*_x_ = 1.678 Mg m^−^^3^
Monoclinic, *P*2_1_/*n*	Mo *K*α radiation, λ = 0.71073 Å
Hall symbol: -P 2yn	Cell parameters from 1433 reflections
*a* = 6.9139 (16) Å	θ = 2.6–25.1°
*b* = 4.0931 (10) Å	µ = 4.58 mm^−^^1^
*c* = 31.480 (7) Å	*T* = 298 K
β = 95.186 (3)°	Block, yellow
*V* = 887.2 (4) Å^3^	0.15 × 0.14 × 0.14 mm
*Z* = 2	

### Data collection

**Table:**

Bruker SMART CCD area-detector diffractometer	1308 reflections with *I* > 2σ(*I*)
Radiation source: fine-focus sealed tube	*R*_int_ = 0.035
graphite	θ_max_ = 25.5°, θ_min_ = 2.6°
φ and ω scans	*h* = −8→8
4238 measured reflections	*k* = −4→4
1627 independent reflections	*l* = −22→38

### Refinement

**Table:**

Refinement on *F*^2^	Primary atom site location: structure-invariant direct methods
Least-squares matrix: full	Secondary atom site location: difference Fourier map
*R*[*F*^2^ > 2σ(*F*^2^)] = 0.034	Hydrogen site location: inferred from neighbouring sites
*wR*(*F*^2^) = 0.081	H-atom parameters constrained
*S* = 1.04	*w* = 1/[σ^2^(*F*_o_^2^) + (0.0347*P*)^2^ + 0.331*P*] where *P* = (*F*_o_^2^ + 2*F*_c_^2^)/3
1627 reflections	(Δ/σ)_max_ < 0.001
110 parameters	Δρ_max_ = 0.36 e Å^−^^3^
0 restraints	Δρ_min_ = −0.37 e Å^−^^3^

### Special details

**Table:**

Geometry. All esds (except the esd in the dihedral angle between two l.s. planes) are estimated using the full covariance matrix. The cell esds are taken into account individually in the estimation of esds in distances, angles and torsion angles; correlations between esds in cell parameters are only used when they are defined by crystal symmetry. An approximate (isotropic) treatment of cell esds is used for estimating esds involving l.s. planes.
Refinement. Refinement of *F*^2^ against ALL reflections. The weighted *R*-factor wR and goodness of fit *S* are based on *F*^2^, conventional *R*-factors *R* are based on *F*, with *F* set to zero for negative *F*^2^. The threshold expression of *F*^2^ > σ(*F*^2^) is used only for calculating *R*-factors(gt) etc. and is not relevant to the choice of reflections for refinement. *R*-factors based on *F*^2^ are statistically about twice as large as those based on *F*, and *R*- factors based on ALL data will be even larger.

### Fractional atomic coordinates and isotropic or equivalent isotropic displacement parameters (Å^2^)

**Table:**

	*x*	*y*	*z*	*U*_iso_*/*U*_eq_	
Br1	1.09932 (5)	0.94173 (10)	0.208298 (11)	0.05710 (17)	
C1	0.2315 (5)	0.7300 (10)	−0.01605 (10)	0.0558 (9)	
H1A	0.2938	0.9047	0.0003	0.084*	
H1B	0.1611	0.8175	−0.0412	0.084*	
H1C	0.3279	0.5791	−0.0242	0.084*	
C2	0.0949 (4)	0.5575 (8)	0.01014 (10)	0.0424 (8)	
C3	0.3412 (5)	0.5400 (8)	0.10658 (10)	0.0459 (8)	
H3	0.2486	0.4244	0.1201	0.055*	
C4	0.5231 (4)	0.6363 (8)	0.13083 (9)	0.0384 (7)	
C5	0.5589 (5)	0.5517 (8)	0.17327 (10)	0.0445 (8)	
H5	0.4663	0.4334	0.1865	0.053*	
C6	0.7302 (4)	0.6397 (8)	0.19646 (10)	0.0435 (8)	
H6	0.7533	0.5825	0.2251	0.052*	
C7	0.8655 (4)	0.8131 (8)	0.17650 (10)	0.0399 (7)	
C8	0.8342 (5)	0.9003 (8)	0.13405 (10)	0.0470 (8)	
H8	0.9278	1.0161	0.1208	0.056*	
C9	0.6625 (4)	0.8128 (9)	0.11179 (10)	0.0467 (8)	
H9	0.6392	0.8733	0.0833	0.056*	
N1	0.1261 (4)	0.4971 (7)	0.05018 (9)	0.0492 (8)	
N2	0.3081 (4)	0.6106 (8)	0.06790 (8)	0.0545 (8)	

### Atomic displacement parameters (Å^2^)

**Table:**

	*U*^11^	*U*^22^	*U*^33^	*U*^12^	*U*^13^	*U*^23^
Br1	0.0437 (2)	0.0655 (3)	0.0587 (3)	−0.00573 (17)	−0.01464 (16)	−0.00470 (18)
C1	0.0459 (18)	0.072 (3)	0.049 (2)	−0.0148 (19)	−0.0034 (16)	0.0041 (19)
C2	0.0395 (17)	0.047 (2)	0.0392 (18)	−0.0009 (14)	−0.0035 (14)	−0.0010 (15)
C3	0.0399 (17)	0.057 (2)	0.0395 (19)	−0.0073 (15)	−0.0008 (14)	0.0015 (15)
C4	0.0382 (16)	0.044 (2)	0.0314 (16)	−0.0019 (14)	−0.0034 (13)	−0.0031 (13)
C5	0.0443 (17)	0.052 (2)	0.0373 (18)	−0.0060 (15)	0.0032 (14)	0.0012 (15)
C6	0.0483 (18)	0.052 (2)	0.0289 (16)	−0.0012 (15)	−0.0048 (14)	0.0032 (14)
C7	0.0332 (15)	0.0444 (19)	0.0404 (18)	0.0015 (14)	−0.0072 (13)	−0.0091 (15)
C8	0.0446 (18)	0.057 (2)	0.0388 (19)	−0.0132 (16)	0.0029 (15)	0.0024 (16)
C9	0.0497 (18)	0.059 (2)	0.0300 (16)	−0.0081 (17)	−0.0019 (14)	0.0046 (15)
N1	0.0407 (15)	0.065 (2)	0.0397 (16)	−0.0119 (13)	−0.0084 (12)	0.0001 (13)
N2	0.0466 (16)	0.075 (2)	0.0387 (16)	−0.0154 (14)	−0.0105 (13)	0.0030 (14)

### Geometric parameters (Å, °)

**Table:**

Br1—C7	1.898 (3)	C4—C9	1.384 (4)
C1—C2	1.487 (4)	C5—C6	1.382 (4)
C1—H1A	0.9600	C5—H5	0.9300
C1—H1B	0.9600	C6—C7	1.371 (4)
C1—H1C	0.9600	C6—H6	0.9300
C2—N1	1.283 (4)	C7—C8	1.381 (4)
C2—C2^i^	1.483 (6)	C8—C9	1.371 (4)
C3—N2	1.252 (4)	C8—H8	0.9300
C3—C4	1.465 (4)	C9—H9	0.9300
C3—H3	0.9300	N1—N2	1.408 (4)
C4—C5	1.381 (4)		
			
C2—C1—H1A	109.5	C4—C5—H5	119.5
C2—C1—H1B	109.5	C6—C5—H5	119.5
H1A—C1—H1B	109.5	C7—C6—C5	118.7 (3)
C2—C1—H1C	109.5	C7—C6—H6	120.6
H1A—C1—H1C	109.5	C5—C6—H6	120.6
H1B—C1—H1C	109.5	C6—C7—C8	121.6 (3)
N1—C2—C2^i^	115.2 (4)	C6—C7—Br1	119.1 (2)
N1—C2—C1	125.2 (3)	C8—C7—Br1	119.3 (2)
C2^i^—C2—C1	119.6 (3)	C9—C8—C7	118.7 (3)
N2—C3—C4	121.2 (3)	C9—C8—H8	120.7
N2—C3—H3	119.4	C7—C8—H8	120.7
C4—C3—H3	119.4	C8—C9—C4	121.3 (3)
C5—C4—C9	118.6 (3)	C8—C9—H9	119.3
C5—C4—C3	120.5 (3)	C4—C9—H9	119.3
C9—C4—C3	120.9 (3)	C2—N1—N2	113.0 (3)
C4—C5—C6	121.1 (3)	C3—N2—N1	112.8 (3)
			
N2—C3—C4—C5	−178.8 (3)	Br1—C7—C8—C9	−178.4 (3)
N2—C3—C4—C9	1.0 (5)	C7—C8—C9—C4	−1.0 (5)
C9—C4—C5—C6	−0.1 (5)	C5—C4—C9—C8	0.7 (5)
C3—C4—C5—C6	179.7 (3)	C3—C4—C9—C8	−179.1 (3)
C4—C5—C6—C7	−0.2 (5)	C2^i^—C2—N1—N2	179.9 (3)
C5—C6—C7—C8	−0.1 (5)	C1—C2—N1—N2	−0.3 (5)
C5—C6—C7—Br1	178.9 (2)	C4—C3—N2—N1	179.9 (3)
C6—C7—C8—C9	0.7 (5)	C2—N1—N2—C3	−177.4 (3)

Symmetry codes: (i) −*x*, −*y*+1, −*z*.
